# Association between Chronic Obstructive Pulmonary Disease and Lung Cancer: A Case-Control Study in Southern Chinese and a Meta-Analysis

**DOI:** 10.1371/journal.pone.0046144

**Published:** 2012-09-28

**Authors:** Hui Wang, Lei Yang, Linnan Zou, Dongsheng Huang, Yuan Guo, Mingan Pan, Yigang Tan, Haibo Zhong, Weidong Ji, Pixin Ran, Nanshan Zhong, Jiachun Lu

**Affiliations:** 1 School of Public Health, The Institute for Chemical Carcinogenesis, The State Key Lab of Respiratory Disease, Guangzhou Medical University, Guangzhou, Guangdong, China; 2 Guangzhou Institute of Respiratory Diseases, The First Affiliated Hospital, The State Key Lab of Respiratory Disease, Guangzhou Medical University, Guangzhou, Guangdong, China; 3 Department of Respiratory Medicine, Guangzhou Chest Hospital, Guangzhou, Guangdong, China; 4 The Third Affiliated Hospital of Guangzhou Medical University, Guangzhou, Guangdong, China; 5 Department of Respiratory Medicine, The Third Affiliated Hospital of Sun Yat-sen University, Guangzhou, Guangdong, China; 6 Department of Respiratory Medicine, Guangzhou Red Cross Hospital, Guangzhou, Guangdong, China; Clinica Universidad de Navarra, Spain

## Abstract

**Background:**

Lung cancer and chronic obstructive pulmonary disease (COPD) share a common risk factor in cigarette smoking and a large portion of patients with lung cancer suffer from COPD synchronously. We therefore hypothesized that COPD is an independent risk factor for lung cancer. Our aim was to investigate the intrinsic linkage of COPD (or emphysema, chronic bronchitis and asthma) and lung cancer.

**Methods:**

The present hospital-based case-control study included 1,069 patients with newly diagnosed lung cancer and 1,132 age frequency matched cancer-free controls. The odds ratios (ORs) for the associations between each previous pulmonary disease and lung cancer were estimated with logistic regression models, adjusting for age, sex, family history of cancer, BMI and pack year smoking. In meta-analysis, the pooled effects of previous pulmonary diseases were analyzed with random effects models; and stratification analyses were conducted on smoking status and ethnicity.

**Results:**

In the case-control study, previous COPD was associated with the odds for increased risk of lung cancer (OR = 1.29, 95% confidence interval [CI] = 1.00∼1.68); so were emphysema (OR = 1.55, 95%CI = 1.03∼2.32) and chronic bronchitis (OR = 1.22, 95%CI = 0.99∼1.67); while asthma was associated with odds for decreased risk of lung cancer (OR = 0.29, 95%CI = 0.16∼0.53). These associations were more pronounced in smokers (*P*<.05 for all strata), but not in non-smokers. In meta-analysis, 35 studies (22,010 cases and 44,438 controls) were identified. COPD was significantly associated with the odds for increased risk of lung cancer (pooled OR = 2.76; 95% CI = 1.85–4.11), so were emphysema (OR = 3.02; 95% CI = 2.41–3.79) and chronic bronchitis (OR = 1.88; 95% CI = 1.49–2.36); and these associations were more pronounced in smokers than in non-smokers (*P*<.001 respectively). No significant association was observed for asthma.

**Conclusion:**

Previous COPD could increase the risk of lung cancer, especially in smokers.

## Introduction

Lung cancer and chronic obstructive pulmonary disease (COPD) are the leading causes of morbidity and mortality worldwide [1]. Lung cancer is the most frequent cancer and the leading cause of cancer related deaths [2]; its five-year survival rate is next to the lowest of all cancers [3].The chronic obstructive pulmonary disease (COPD) has been another major health problem all over the world with increasing incidence. In China, its prevalence was 8.2% among adults >40 years old; and in Chinese rural areas, COPD has ever been ranked the third leading cause of death [4]. Because COPD is a group of pulmonary diseases characterized by non-reversible airflow limitation and developing over time [5], it has usually been referred to be other common pulmonary diseases with respiratory limitation such as emphysema, chronic bronchitis, asthma or bronchiectasis in clinic.

It is well recognized that smoke exposure causes both lung cancer and COPD [6]–[7]. Some epidemiologic studies found that smokers with COPD are up to five-fold more susceptible to lung cancer than smokers with normal lung function [8]. Furthermore, reduced lung function is an important feature in the development of lung cancer, and forced expiratory in one second (FEV1) has been proven a biomarker of wider respiratory risk from smoking [9], suggesting that there might be an association between COPD and lung cancer. Recent studies have found that tobacco smoke stimulates both local and systemic inflammation, which may play a causal role in both lung cancer and COPD [10]. As COPD and other chronic pulmonary diseases such as emphysema, chronic bronchitis and asthma could be caused by inflammation in lung tissues, these conditions may act as intermediates or catalysts in the development of lung neoplasms or be related with lung cancer development through common etiologies and/or exposures [11], [12]. However, the causal relationship between COPD and lung tumorigenesis is not yet fully understood. Based on information we obtained, we hypothesized that COPD might be an independent risk factor for lung cancer.

In order to investigate the association between COPD and the risk of lung cancer, we conducted a case-control study including patients with newly diagnosed lung cancer and age frequency matched cancer-free controls in Southern Chinese during March 2007 to April 2010, and a meta-analysis including 35 studies. The effects of other pulmonary diseases and tobacco smoking on lung cancer were also analyzed in the stratifications. Our study will provide new evidence that COPD is a risk factor in lung cancer development.

## Methods

### Ethics statement

Informed consent was obtained from each subject before study participation. The study was approved by the institutional review boards of Guangzhou Medical University.

### Case-control study

#### Study subjects

We conducted a hospital-based case-control study of total 1069 patients with newly diagnosed lung cancer and 1132 age frequency matched cancer-free controls. As described previously [13]–[16], all subjects were genetically unrelated ethnic Han Chinese from Guangzhou City and surrounding regions in Southern China. Patients with histopathologically confirmed diagnosis of lung cancer were consecutively recruited between March 2007 and March 2010 at the urban hospitals (i.e. the First, the Second and the Tumor Hospitals affiliated to Guangzhou Medical Hospital and the Guangzhou Chest Hospital) and at the suburb of Guangzhou city (i.e. Panyu People's Hospital), with a response rate of ∼95%. The cancer-free controls were from a subject pool of more than 10,000 individuals who participated in healthy checkup programs in the community health stations in Guangzhou City during the same time period when the cases were recruited. Among them, controls with frequency matched to the cases by age (±5 years) were randomly selected with a response rate of ∼85% [13]–[16].

#### Data collection

In-person interviews were conducted for all cases and controls by blinding-trained interviewers using a structured questionnaire. Detailed questions were asked about smoking status, pack-year smoking, alcohol use and other factors including family history of cancer, menstrual history, sex, age and BMI (See **Protocol S1** for more details). Cases and controls were also asked whether a physician had ever informed that they had specific lung diseases (i.e., COPD, emphysema, chronic bronchitis and asthma), and if they had, they were asked to provide detailed medical diagnosis evidences for these diseases. All the subjects with COPD were diagnosed with spirometry. For other diseases like emphysema, chronic bronchitis or asthma, their diagnoses were mainly based on self-report. Only individuals with COPD (emphysema, chronic bronchitis or asthma) diagnosed more than 1 year before lung cancer diagnosis for cases or enrollment for controls were considered to have pre-existing lung diseases, otherwise those who provided a date of previous COPD (or chronic bronchitis, emphysema, and asthma) less than one year or lacked medical evidences were excluded. In addition, emphysema, chronic bronchitis and asthma are closely related with COPD; those emphysema, chronic bronchitis or asthma patients who have an irreversible limitation of the flow of air are COPD patients, but not vice versa. The detailed diagnosis standard for these lung diseases were described in Appendix (**Protocol S1**).

### Meta analysis

#### Literature search strategy

We conducted a literature search using Cochrane library, Pub med and China National Knowledge Infrastructure (CNKI) databases as well as traced citations from January 1980 to December 2010 to obtain a plenty of published or on-line articles about the association between previous COPD (or emphysema, chronic bronchitis, and asthma) and lung cancer. We utilized the Medical Subject Headings “COPD” or “chronic obstructive pulmonary disease” or “emphysema” or “chronic bronchitis” or “respiratory tract diseases” or “asthma” or “lung diseases” and “lung neoplasm” or the key word terms “previous lung disease” or “risk” and “lung cancer”. Language was limited to English and Chinese. Titles and abstracts were reviewed for article relevance.

#### Study selection and data extraction

**Figure 1:**
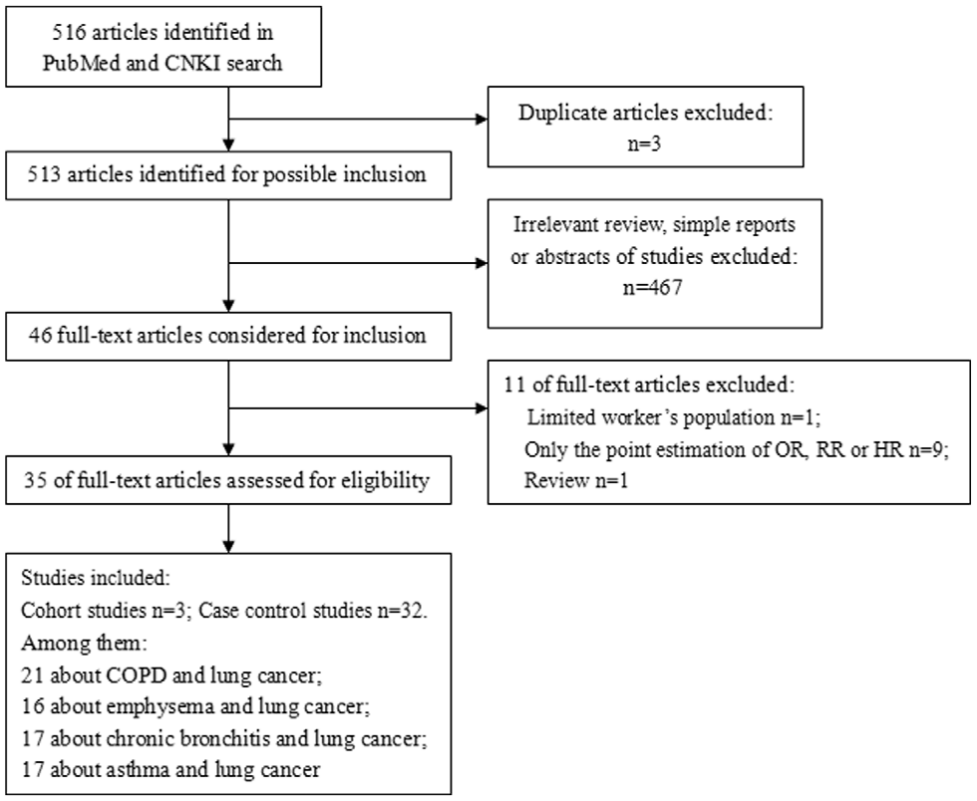
Flowchart of selection of studies for inclusion in the meta-analysis.

We complied with the principle of Moose [17] to evaluate quality of studies. When the same population was examined in multiple publications, we included only the estimate with the largest number of cases reported. The studies about occupational subjects were excluded in order to avoid the selection bias. **Figure 1** shows the process of selection of studies [18].

Effect estimates of all identified studies should comprise odds ratio (OR), relative risk (RR) and their corresponding 95% confidence intervals. Extracted information contained publication date, age of subjects, sex, race, smoking status (current smokers, former smokers and non-smokers), diagnostic methods of COPD and lung cancer, histological type of lung cancer and exposure rate or incidence rate of two groups. Information above were collected and evaluated for quality by two researchers respectively and then cross-checked.

### Statistical methods

For the case-control study, comparisons of dichotomous risk factors between cases and controls were conducted using χ^2^ tests; comparisons of means were conducted using *t* tests. The association between each previous pulmonary disease (COPD, emphysema, chronic bronchitis, and asthma) and lung cancer, measured by the odds ratio (OR) and its corresponding 95% confidence interval (CI), was estimated using unconditional logistic regression, without or with adjustment for age, sex, family history of cancer, BMI and pack year smoking. The data were further stratified by smoking status to evaluate the stratum variable-related ORs. The difference between associations in smokers and those in overall individuals were tested as Douglas G Altman et al. suggested [19]. Homogeneity between strata variable-related ORs was tested. Two-sided tests were used with *P*≤.05 considered statistically significant. All statistical analyses were performed using SPSS version 13.0.

For the meta-analysis, the data were stratified by smoking status (smokers, non-smokers and not-identified) and ethnicity (Caucasian and Asian) to evaluate the stratum variable-related ORs for the associations between each previous pulmonary disease (COPD, emphysema, chronic bronchitis, and asthma) and the risk of lung cancer. Pooled ORs and 95%CIs were used as the measure of effect estimates for all studies. The test statistic Q for heterogeneity was calculated and the null hypothesis that all studies in each stratum are homogeneous is rejected. Thus, the random effects model was adopted. Sensitivity analysis was utilized to test stability of the results. Publication bias was determined by funnel plots. Analysis was conducted through DerSimonian-Laird method using Rev Man 5.0 software provided by the Cochrane Collaboration.

## Results

### Case-control study

There were 1069 patients with newly diagnosed lung cancer and 1132 age frequency matched cancer-free controls. The distributions of demographic characteristics of study population are shown in **Table 1**. The average ages were 60.3±12.3 and 60.5±12.3 years old respectively in cases and controls, and there was no significant difference in age, family history of cancer and drinking (*P* = .90, .42 and .64, respectively) in the two groups. However, the cases had lower education level, lower BMI, were more likely to be current and ex-smokers, and had more prevalence of COPD, emphysema, chronic bronchitis and asthma than controls.

**Table 1:** Characteristics of lung cancer cases and controls in Southern Chinese.

Characteristic	Cases n (%)	Controls n (%)	*P* Value
**Total no.**	**1069**	**1132**	
Age(years)			.901
≤60	537(50.2)	572(50.5)	
>60	532(49.8)	560(49.5)	
Age (means±SD)	60.3±12.3	60.5±12.3	.797
Sex (%)			.012
Male	758(70.9)	746(65.9)	
Female	311(29.1)	386(34.1)	
Education level (%)			<.001
≤ primary school	410(42.6)	378(35.6)	
Junior middle school	318(33.0)	303(28.5)	
Senior middle school	137(14.2)	292(27.5)	
≥ college	98(10.2)	90(8.5)	
Family history of cancer (%)			.416
Yes	104(9.7)	98(8.7)	
No	965(90.3)	1033(91.3)	
BMI (kg/m^2^) (means±SD)^a^	21.3±3.3	23.3±3.6	<.001
BMI≤23.9	898(84.0)	693(61.7)	<.001
24.0∼27.9	146(13.7)	325(28.9)	
≥28.0	25(2.3)	105(9.3)	

a : BMI, body mass index.

**Table 2** shows the associations between previous pulmonary diseases, tobacco smoking or alcohol exposure and the risk of lung cancer. Previous COPD, emphysema, and chronic bronchitis were significantly associated with the odds for increased risk of lung cancer in univariate analysis. However, there was inversely association between asthma and lung cancer. After adjusted by age, sex, family history of cancer, BMI and pack year smoking, the significantly associations with lung cancer were still seen for previous COPD (OR: 1.29, 95%CI: 1.00–1.68), emphysema (OR: 1.55, 95% CI: 1.03–2.32), chronic bronchitis (OR: 1.22, 95%CI: 0.99–1.67, boundary statistical significance) and asthma (OR: 0.29, 95%CI: 0.16–0.53).

**Table 2:** Association between previous pulmonary diseases, tobacco smoking or alcohol exposure and the risk of lung cancer in Southern Chinese.

Characteristic	Cases (n = 1069) n (%)	Controls (n = 1132) n (%)	Crude OR (95% CI)	Adjusted OR^a^ (95% CI)
COPD				
Yes	187(17.5)	136(12.1)	1.54 (1.21∼1.96)	1.29(1.00∼1.68)
No	882(82.5)	996(87.9)	1.00	1.00
Emphysema				
Yes	77(7.2)	44(3.9)	1.92(1.31∼2.81)	1.55(1.03∼2.32)
No	992(92.8)	1087(96.1)	1.00	1.00
Chronic bronchitis				
Yes	118(11.0)	89(7.9)	1.45(1.09∼1.94)	1.22(0.99∼1.67)
No	951(89.0)	1042(92.1)	1.00	1.00
Asthma				
Yes	15(1.4)	45(4.0)	0.34(0.19∼0.62)	0.29(0.16∼0.53)
No	1054(98.6)	1085(96.0)	1.00	1.00
Smoking status				
Current and ex-smokers	609(57.0)	542(47.9)	1.44 (1.22∼1.71)	1.39(1.11∼1.73)
Non-smokers	460(43.0)	590(52.1)	1.00	1.00
Pack year smoking				
≥20	465(43.7)	314(30.2)	1.66(1.37∼2.00)	1.79(1.40∼2.30)
<20	139(13.1)	211(20.3)	0.74(0.57∼0.94)	0.83(0.62∼1.11)
0	460(43.2)	514(49.5)	1.00	1.00
Drinking status				
Current and ex-drinkers	230(21.5)	234(20.7)	1.05(0.86∼1.29)	1.01(0.81∼1.26)
Non-drinkers	839(78.5)	898(79.3)	1.0	1.0

a adjusted by age, sex, family history of cancer,, BMI and pack year smoking in the multivariate logistic regression analysis.

**Table 3** presented stratified results on smoking status for the associations between four previous pulmonary diseases and lung cancer. Among current and ex-smokers, previous COPD and emphysema was significantly associated with the odds for increased risk of lung cancer (OR: 1.48, 95% CI: 1.08–2.05 for COPD; OR: 1.74, 95% CI: 1.06–2.87 for emphysema). Such significant association was not seen for chronic bronchitis (OR: 1.32, 95% CI: 0.91–1.92, *P* = .14). However, previous asthma was significantly associated with the odds for decreased risk of lung cancer (OR: 0.19, 95% CI: 0.08–0.44). Among non-smokers, no significant associations were seen between all the four previous pulmonary diseases and lung cancer.

**Table 3:** Associations of lung cancer with previous pulmonary diseases, stratified by smoking status.

Characteristic	Smokers		OR(95% CI)^b^	Non-smokers		OR(95% CI)^b^
	Cases^a^ (n = 609)	Controls^a^ (n = 542)		Cases^a^ (n = 460)	Controls^a^ (n = 590)	
COPD						
No	463(76.0)	455(83.9)	1.00	419(91.1)	534(91.6)	1.00
Yes	146(24.0)	87(16.1)	1.48(1.08∼2.05)	41(8.9)	49(8.4)	1.00(0.63∼1.59)
Emphysema						
No	548(90.0)	515(95.0)	1.00	444(96.5)	572(97.1)	1.00
Yes	61(10.0)	27(5.0)	1.76 (1.07∼2.90)	16(3.5)	17(2.9)	1.07(0.51∼2.25)
Chronic bronchitis						
No	516(84.7)	481(88.7)	1.00	435(94.6)	561(95.2)	1.00
Yes	93(15.3)	61(11.3)	1.32(0.91∼1.92)	25(5.4)	28(4.8)	1.18(0.66∼2.12)
Asthma						
No	601(98.7)	515(95.0)	1.00	453(98.5)	570(96.9)	1.00
Yes	8(1.3)	27(5.0)	0.19(0.08∼0.44)	7(1.5)	18(3.1)	0.50(0.20∼1.27)

a : Data are presented by n (%);

b : adjusted by gender, age, family history of cancer, and BMI in the multivariate logistic regression analysis.

### Meta analysis

The process of literature selection was showed in **Figure 1**. We finally identified 35 eligible studies to be reviewed quantitatively [20]–[54]. Among these 35 studies, there were 29 case-control studies and 6 cohort studies [21], [27], [32], [33], [35], [37]. There were 22,010 cases and 44,438 controls totally. Twenty three studies were conducted in Caucasian population, 12 were conducted in Asians (one for Japanese, the others for Chinese), and 1 in Africans (not involved in the stratified analysis on ethnicity due to only one study). Characteristics of 35 studies were showed in Table S1. The diagnosis of lung cancer was based on pathology (dominating), computerized tomography (CT) and medical records. The diagnosis of COPD was based on spirometry, questionnaire survey or physician report (**Table S1**). Significant heterogeneity was observed among estimates across all 35 studies as well as among the subgroups when stratified by smoking status and ethnicity.

Among 35 studies, there were 21 studies about COPD and lung cancer, 16 studies about emphysema and lung cancer, 17 studies about chronic bronchitis and lung cancer, and 17 studies about asthma and lung cancer. In terms of disease-specific estimates, the overall ORs for COPD, emphysema, chronic bronchitis and asthma were 2.76 (95% CI: 1.85–4.11), 3.02 (95%CI: 2.41–3.79), 1.88 (95%CI: 1.49–2.36) and 1.02 (95%CI: 0.50–2.10), respectively.

**Figure 2:**
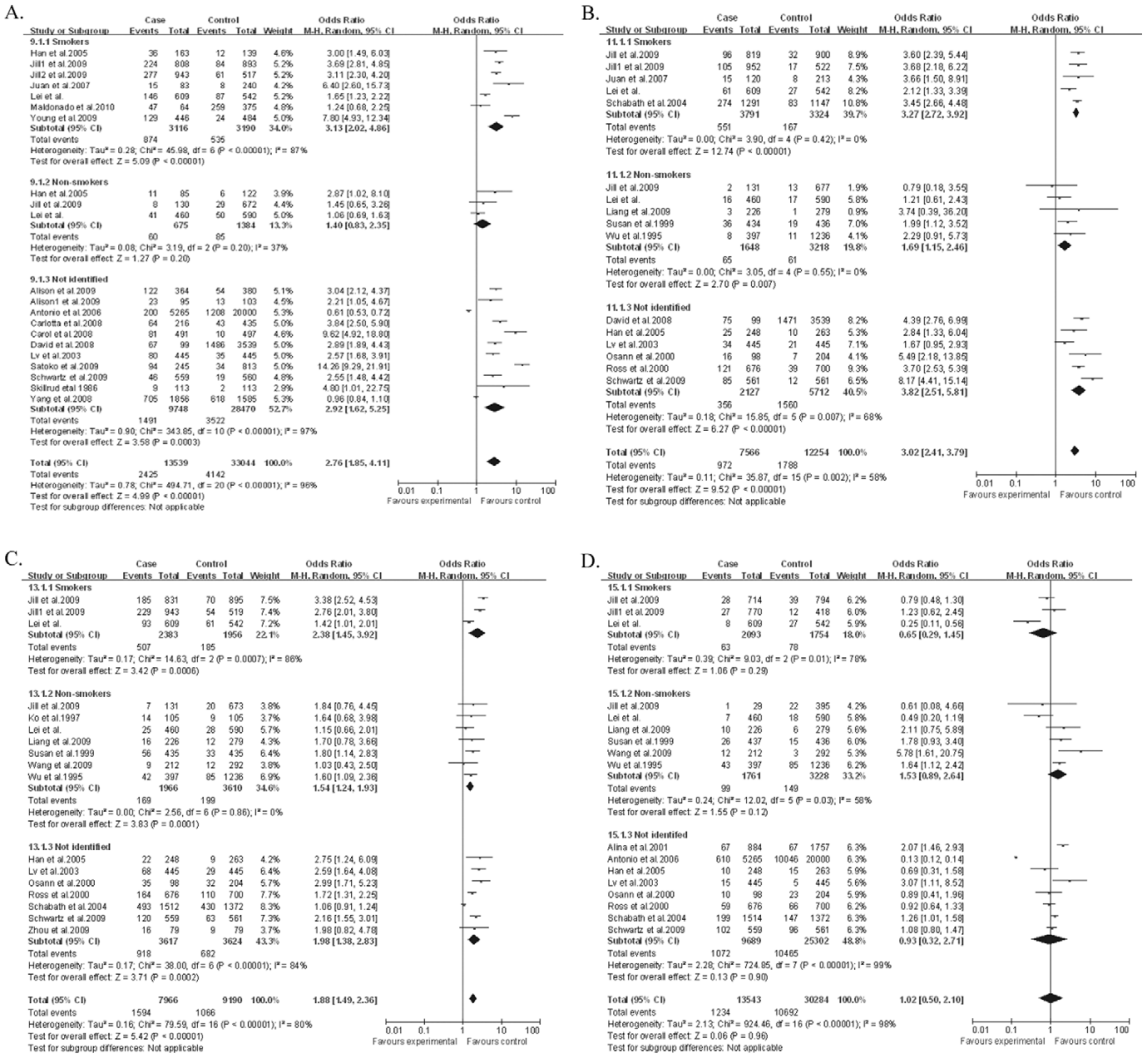
**A.** Pooled effect estimates of the lung cancer risk associated with a previous history of COPD, stratified by smoking status and overall. B. Pooled effect estimates of the lung cancer risk associated with a previous history of emphysema, stratified by smoking status and overall. **C.** Pooled effect estimates of the lung cancer risk associated with a previous history of chronic bronchitis, stratified by smoking status and overall. **D.** Pooled effect estimates of the lung cancer risk associated with a previous history of asthma, stratified by smoking status and overall.

When restricted to smokers, all the associations between COPD, emphysema or chronic bronchitis and lung cancer were stronger than the overall ones when we only saw point estimate of ORs. However, the difference were not statistically significant (*P*>0.05 for all). The ORs for these three previous pulmonary diseases were 3.13 (95% CI: 2.02–4.86), 3.27 (95%CI: 2.72–3.92) and 2.38 (95%CI: 1.45–3.92). Furthermore, the ORs in smokers for these four diseases were higher than those in non-smokers (*P*<.001). Among non-smokers, there were significant associations between emphysema and chronic bronchitis and lung cancer (OR: 1.69, 95%CI: 1.15–2.46 for emphysema; OR: 1.54, 95%CI: 1.24–1.93 for chronic bronchitis), while the association for COPD was not significant (OR: 1.40, 95%CI: 0.83–2.35). For asthma, we did not see a significant association with lung cancer for all subgroups after stratified by smoker status. (**Table 4** **and** **Figure 2**).

**Table 4:** Estimated pooled ORs (95% CI) for the association of four previous pulmonary diseases with lung cancer in meta-analysis, stratified by ethnicity and smoking status.

Characteristic	Smoking status			*P* a Value	Ethnicity		*P* b Value	Total
	Smokers	Non-smokers	Not identified		Caucasian	Asian		
COPD	3.13(2.02,4.86)	1.40(0.83,2.35)	2.92(1.62,5.25)	<.001	2.42(1.46,4.00)	2.98(1.52,5.84)	<.001	2.76(1.85,4.11)
Emphysema	3.27(2.72,3.92)	1.69(1.15,2.46)	3.82(2.51,5.81)	<.001	3.62(2.91,4.50)	1.90(1.42,2.54)	<.001	3.02(2.41,3.79)
Chronic bronchitis	2.38(1.45,3.92)	1.54(1.24,1.93)	1.98(1.38,2.83)	<.001	2.01(1.45,2.79)	1.68(1.31,2.15)	.114	1.88(1.49,2.36)
Asthma	0.65(0.29,1.45)	1.53(0.89,2.64)	0.93(0.32,2.71)	<.001	0.88(0.35,2.21)	1.25(0.59,2.67)	<.001	1.02(0.50,2.10)

a : The comparison of ORs between smokers and non-smokers.

b : The comparison of ORs between Caucasian and Asian.

When stratified by ethnicity, the strong association was still observed in both Asian and Caucasian for COPD, emphysema, and chronic bronchitis, but not for asthma. The relevant data can be seen in **Table 4** **and Figure S1**.

There was no evidence suggestive of publication bias for all associations and separated associations for each previous disease (COPD, emphysema, chronic bronchitis and asthma). All of the *P* values of Begg's tests for different combinations were greater than .05. (The funnel plots were not shown.) We also performed a sensitivity analysis by stratifying the total studies based on the methods of COPD diagnosis and the result was shown in **Figure S2**. The OR in spirometry subgroup were higher than that in questionnaire or physician subgroups (**Figure S2**).

## Discussion

In our large hospital-based case-control study, we found that previous COPD, emphysema and chronic bronchitis were significantly associated with the odds for increased risk of lung cancer, while previous asthma was associated with the odds for decreased risk of lung cancer. The effect estimates ORs of the associations between these diseases and lung cancer in the subgroup of current and ex-smokers were similar to or slightly stronger than those in the overall subjects. However, we did not observe significant association between each studied disease and lung cancer for never smokers.

In our meta-analysis, significantly odds for increased risk of lung cancer were seen for COPD, emphysema and chronic bronchitis among all the examined smoker status subgroups and all the examined ethnic subgroups except for the non-smoker subgroup for COPD (no significant association was seen in this subgroup). The results were consistent with a previous similar systematic review [55]. However, no significant associations between every previous pulmonary disease and lung cancer were seen among non-smokers in our case-control study. This difference might be because of the insufficient sample size of our case-control study and the existence of some residual bias.

Our results of case-control study were consistent with those of a number of prior studies [31], [37], [46], [54] and reconfirmed the strong association of COPD with lung cancer as well as our meta-analysis. Given the significant association existing only in the smokers, these two diseases may share a common environmental risk factor of cigarette smoke exposure and similar pathogenic mechanisms. In addition, recent studies have suggested a central role of chronic inflammation in the pathogenesis of both diseases [10], [56], [57]. Therefore, immune dysfunction, abnormal activation of NF-kB, epithelial-to-mesenchymal transition (EMT), altered adhesion signaling pathways, and extracellular matrix degradation/altered signaling might be the key underlying mechanisms in both COPD and lung cancer[12].

We speculated that there may be some genetic predisposition to these diseases as the incidence of these diseases was only observed in a fraction of smokers [1]. Genetic variants with a reduced risk of COPD in genome-wide association studies (protective variants on chromosomes 4q31 and 4q22) are independently associated with reduced risk of lung cancer [58]. A number of candidate single gene polymorphisms have been associated with susceptibility to lung cancer and COPD [59]–[62]. However, uncoupling shared risk factors from causal pathways will be difficult and further exploring in molecular and genetic mechanisms of links between COPD and lung cancer is necessary.

Both chronic bronchitis and emphysema can lead to chronic airway obstruction and then develop into COPD, which was often considered as a complex containing chronic bronchitis and emphysema. Our finding also showed that emphysema and chronic bronchitis were risk factors for the development of lung cancer. A positive association of emphysema and chronic bronchitis with lung cancer has been reported in several case-control and cohort studies [32], [36], [63]–[65]. This association was reported mainly among smokers or more pronounced among smokers than non-smokers [20], [31], [32], [46], [63], which are also confirmed by the results of our meta-analysis, suggesting the effect of confounding by smoking. Because the pulmonary impairments caused by these two diseases may reduce the clearance of carcinogens and damaged pulmonary tissue is more susceptible to the effects of carcinogens [66], patients may develop inherent susceptibility to both lung cancer and obstructive lung diseases [67].

Our case-control study also found a significant inverse association between previous asthma and lung cancer, but this association was lost in non-smokers. Controversial findings have been reported on the association between asthma and lung cancer, and a previous meta-analysis study which included seven cohort studies found that asthma was associated with a modest increased risk of lung cancer [68], which was contrary to our finding. However, here we also performed a meta-analysis on this topic, we found that asthma is not significantly associated with lung cancer after combined eight studies, and we also showed asthma may exert inverse effect on modulating lung cancer risk in smokers and never-smokes. A previous study had hypothesized several potential explanations for an inverse association between asthma and lung cancer [69]. Asthmatics might avoid smoking and other deleterious exposures that could trigger their asthma symptoms, which may subsequently reduce their risk of lung cancer. They are also administered allergy medications for a long period of time due to the chronic nature of asthma. Although the potential impact of these medications on lung carcinogenesis is unclear, some of them, for example, antibiotics, might eliminate pathogens related to the development of lung cancer [70].

The present study has a number of strengths. It combined an original observational study (case-control study) and a quantitative review (meta-analysis). The sample size of the case-control study was large, and the questionnaires were administered by interviewers who underwent centralized training, ensuring that important demographic and risk factor information (e.g. age, smoking) was obtained as accurately and completely as possible. In the meta- analysis, we evaluated the associations between four previous pulmonary diseases (COPD, emphysema, chronic bronchitis and asthma) and lung cancer respectively. It is worth mentioned that for each disease, we not only calculated combined estimate of the overall subjects, but also calculated separate pooled estimates of the subgroups stratified by smoking status and ethnicity, while the previous meta-analyses about this topic did no use stratification based on smoking status or ethnicity. In addition, publication bias was not evident in our study, which indicated a good representation of the extracted studies.

There are several design and methodological limitations of this study that must be considered in the analysis and interpretation of the results. First, as an inherent limitation for case-control study, information bias was inevitable. The definitions of emphysema, chronic bronchitis and asthma through self-report would lead to misclassification bias. The cases might have higher report rates of the events of previous pulmonary diseases than the controls due to their caring more about their physical conditions (recall bias). To minimize information bias, subjects were asked only about a specific list of lung diseases, which had to be diagnosed by a physician or have resulted in treatment and/or hospitalization. In the meta-analysis, misclassification bias also existed due to the different definitions of chronic pulmonary diseases in different studies (e.g., self report or spirometry test, etc). Then we performed a sensitivity analysis by stratifying the total studies based on the methods of COPD diagnosis and found that the value of OR in spirometry subgroup were higher than that in questionnaire or physician subgroups, which indicated that recall bias about COPD could underestimate its effect on the risk for lung cancer. Next, the latency analyses lacked in our study. Examining the time of onset of previous lung diseases prior to cancer diagnosis could identify the reverse causality and obtain more accurate estimates of association. The third limitation is that residual confounding existed in our study because some confounding factors were not considered (e.g. biofuel smoke and passive smoke). Further investigations should supplement the variables about biofuel smoke and passive smoke to assess the magnitude of residual confounding.

In conclusion, our study verified the associations of COPD, emphysema, and chronic bronchitis with the increased risk of lung cancer. The association between asthma and lung cancer was still uncertain. Our extensive analyses of these associations by smoking status suggested that smoking is a shared risk factor of both COPD and lung cancer. There may be a central role of chronic inflammation in their pathogenesis in addition to some shared genetic predisposition to the two diseases. Further investigations into the mechanisms for relevance of COPD with the development of lung cancer are warranted. Moreover, because smoking habit varies largely between different genders and estrogen and autoimmunity were possibly associated with lung diseases [71], further investigations in studies powered to evaluate men and women separately might help to identify more details in the pathogenesis of lung cancer.

## Supporting Information

Protocol S1 **Additional details of methods.**
(DOC)

Table S1 **Characteristics of 35 studies on the association between COPD and lung cancer.**
(DOC)

Figure S1 **A.**Pooled effect estimates of the lung cancer risk associated with a previous history of COPD, separated by ethnicity and overall. **B.** Pooled effect estimates of the lung cancer risk associated with a previous history of emphysema, separated by ethnicity and overall. **C.** Pooled effect estimates of the lung cancer risk associated with a previous history of chronic bronchitis, separated by ethnicity and overall. **D.** Pooled effect estimates of the lung cancer risk associated with a previous history of asthma, separated by ethnicity and overall.
(TIF)

Figure S2 **Pooled effect estimates of the lung cancer risk associated with a previous history of COPD, stratified by method of COPD diagnosis and overall (sensitivity analysis).**
(TIF)

## References

[1] Punturieri A, Szabo E, Croxton TL, Shapiro SD, Dubinett SM (2009) Lung cancer and chronic obstructive pulmonary disease: needs and opportunities for integrated research. J Natl Cancer Inst 101: 554–559. 19351920 10.1093/jnci/djp023 PMC2669099

[2] Jemal A, Siegel R, Xu J, Ward E (2010) Cancer statistics, 2010. CA Cancer J Clin 60: 277–300. 20610543 10.3322/caac.20073

[3] Mathers CD, Loncar D (2006) Projections of global mortality and burden of disease from 2002 to 2030. PLoS Med 3: e442. 17132052 10.1371/journal.pmed.0030442 PMC1664601

[4] Zhong N, Wang C, Yao W, Chen P, Kang J, et al (2007) Prevalence of chronic obstructive pulmonary disease in China: a large, population-based survey. Am J Respir Crit Care Med 176: 753–760. 17575095 10.1164/rccm.200612-1749OC

[5] Rabe KF, Hurd S, Anzueto A, Barnes PJ, Buist SA, et al (2007) Global strategy for the diagnosis, management, and prevention of chronic obstructive pulmonary disease: GOLD executive summary. Am J Respir Crit Care Med 176: 532–555. 17507545 10.1164/rccm.200703-456SO

[6] Caramori G, Casolari P, Cavallesco GN, Giuffre S, Adcock I, et al (2011) Mechanisms involved in lung cancer development in COPD. Int J Biochem Cell Biol 43: 1030–1044. 20951226 10.1016/j.biocel.2010.08.022

[7] Adcock IM, Caramori G, Barnes PJ (2011) Chronic obstructive pulmonary disease and lung cancer: new molecular insights. Respiration 81: 265–284. 21430413 10.1159/000324601

[8] Young RP, Hopkins RJ, Christmas T, Black PN, Metcalf P, et al (2009) COPD prevalence is increased in lung cancer, independent of age, sex and smoking history. Eur Respir J 34: 380–386. 19196816 10.1183/09031936.00144208

[9] Young RP, Hopkins R, Eaton TE (2007) Forced expiratory volume in one second: not just a lung function test but a marker of premature death from all causes. Eur Respir J 30: 616–622. 17906084 10.1183/09031936.00021707

[10] Petty TL (2005) Are COPD and lung cancer two manifestations of the same disease? Chest 128: 1895–1897. 16236829 10.1378/chest.128.4.1895

[11] Rutgers SR, Postma DS, Ten HN, Kauffman HF, van Der Mark TW, et al (2000) Ongoing airway inflammation in patients with COPD who Do not currently smoke. Chest 117: 262S. 10.1378/chest.117.5_suppl_1.262s 10843943

[12] Yao H, Rahman I (2009) Current concepts on the role of inflammation in COPD and lung cancer. Curr Opin Pharmacol 9: 375–383. 19615942 10.1016/j.coph.2009.06.009 PMC2730975

[13] Liu B, Chen D, Yang L, Li Y, Ling X, et al (2010) A functional variant (−1304T>G) in the MKK4 promoter contributes to a decreased risk of lung cancer by increasing the promoter activity. Carcinogenesis 31: 1405–1411. 20554746 10.1093/carcin/bgq126

[14] Lu J, Yang L, Zhao H, Liu B, Li Y, et al (2011) The polymorphism and haplotypes of PIN1 gene are associated with the risk of lung cancer in Southern and Eastern Chinese populations. Hum Mutat 32: 1299–1308. 21850685 10.1002/humu.21574

[15] Yang L, Li Y, Cheng M, Huang D, Zheng J, et al (2011) A functional polymorphism at microRNA-629 binding site in the 3′-untranslated region of NBS1 gene confers an increased risk of lung cancer in Southern and Eastern Chinese population. Carcinogenesis 33: 338–347. 22114071 10.1093/carcin/bgr272

[16] Liu Bin, Yang Lei, Huang Binfang, Cheng Mei, Wang Hui, et al (2012) A Functional Copy-Number Variation in MAPKAPK2 Predicts Risk and Prognosis of Lung Cancer. Am J Hum Genet 91: 384–390. 22883146 10.1016/j.ajhg.2012.07.003 PMC3415537

[17] Stroup DF, Berlin JA, Morton SC, Olkin I, Williamson GD, et al (2000) Meta-analysis of observational studies in epidemiology: a proposal for reporting. Meta-analysis Of Observational Studies in Epidemiology (MOOSE) group. JAMA 283: 2008–2012. 10789670 10.1001/jama.283.15.2008

[18] Moher D, Liberati A, Tetzlaff J, Altman DG (2009) Preferred reporting items for systematic reviews and meta-analyses: the PRISMA statement. PLoS Med 6: e1000097. 19621072 10.1371/journal.pmed.1000097 PMC2707599

[19] Altman DG, Bland JM (2003) Interaction revisited: the difference between two estimates. BMJ 326: 219. 12543843 10.1136/bmj.326.7382.219 PMC1125071

[20] Maldonado F, Bartholmai BJ, Swensen SJ, Midthun DE, Decker PA, et al (2010) Are airflow obstruction and radiographic evidence of emphysema risk factors for lung cancer? A nested case-control study using quantitative emphysema analysis. Chest 138: 1295–1302. 20348193 10.1378/chest.09-2567

[21] Skillrud DM, Offord KP, Miller RD (1986) high risk of lung cancer in chronic obstructive pulmonary disease. Annals of internal Medicine 105: 503–507. 3752756 10.7326/0003-4819-105-4-503

[22] Young RP, Hopkins RJ, Hay BA, Epton MJ, Mills GD, et al (2009) A gene-based risk score for lung cancer susceptibility in smokers and ex-smokers. Postgrad Med J 85: 515–524. 19789190 10.1136/pgmj.2008.077107

[23] Yang P, Sun Z, Krowka MJ, Aubry MC, Bamlet WR, et al (2008) Alpha1-antitrypsin deficiency carriers, tobacco smoke, chronic obstructive pulmonary disease, and lung cancer risk. Arch Intern Med 168: 1097–1103. 18504338 10.1001/archinte.168.10.1097 PMC2562773

[24] Kishi K, Gurney JW, Schroeder DR, Scanlon PD, Swensen SJ, et al (2002) The correlation of emphysema or airway obstruction with the risk of lung cancer: a matched case-controlled study. Eur Respir J 19: 1093–1098. 12108862 10.1183/09031936.02.00264202

[25] Galeone C, Pelucchi C, La Vecchia C, Negri E, Bosetti C, et al (2008) Indoor air pollution from solid fuel use, chronic lung diseases and lung cancer in Harbin, Northeast China. Eur J Cancer Prev 17: 473–478. 18714191 10.1097/CEJ.0b013e328305a0b9

[26] Gonzalez-Perez A, Fernandez-Vidaurre C, Rueda A, Rivero E, Garcia RL (2006) Cancer incidence in a general population of asthma patients. Pharmacoepidemiol Drug Saf 15: 131–138. 16287212 10.1002/pds.1163

[27] Mannino DM, Aguayo SM, Petty TL, Redd SC (2003) Low lung function and incident lung cancer in the United States: data From the First National Health and Nutrition Examination Survey follow-up. Arch Intern Med 163: 1475–1480. 12824098 10.1001/archinte.163.12.1475

[28] Mizuno S, Takiguchi Y, Fujikawa A, Motoori K, Tada Y, et al (2009) Chronic obstructive pulmonary disease and interstitial lung disease in patients with lung cancer. Respirology 14: 377–383. 19192220 10.1111/j.1440-1843.2008.01477.x

[29] Van Dyke AL, Cote ML, Wenzlaff AS, Chen W, Abrams J, et al (2009) Cytokine and cytokine receptor single-nucleotide polymorphisms predict risk for non-small cell lung cancer among women. Cancer Epidemiol Biomarkers Prev 18: 1829–1840. 19505916 10.1158/1055-9965.EPI-08-0962 PMC3771080

[30] Etzel CJ, Kachroo S, Liu M, D'Amelio A, Dong Q, et al (2008) Development and validation of a lung cancer risk prediction model for African-Americans. Cancer Prev Res (Phila) 1: 255–265. 19138969 10.1158/1940-6207.CAPR-08-0082 PMC2854402

[31] Schwartz AG, Cote ML, Wenzlaff AS, Van Dyke A, Chen W, et al (2009) Chronic obstructive lung diseases and risk of non-small cell lung cancer in women. J Thorac Oncol 4: 291–299. 19190518 10.1097/JTO.0b013e3181951cd1 PMC2745706

[32] de Torres JP, Bastarrika G, Wisnivesky JP, Alcaide AB, Campo A, et al (2007) Assessing the relationship between lung cancer risk and emphysema detected on low-dose CT of the chest. Chest 132: 1932–1938. 18079226 10.1378/chest.07-1490

[33] Wilson DO, Weissfeld JL, Balkan A, Schragin JG, Fuhrman CR, et al (2008) Association of radiographic emphysema and airflow obstruction with lung cancer. Am J Respir Crit Care Med 178: 738–744. 18565949 10.1164/rccm.200803-435OC PMC2556456

[34] Alavanja MC, Brownson RC, Boice JJ, Hock E (1992) Preexisting lung disease and lung cancer among nonsmoking women. Am J Epidemiol 136: 623–632. 1442729 10.1093/oxfordjournals.aje.a116542

[35] Rong GX, Xiao-ou S, Tang GY (2010) The history of chronic pulmonray diseases and male lung cancer risk: a prospective cohort study in urban Shanghai. Tumor 30: 502–504.

[36] Kreuzer M, Heinrich J, Kreienbrock L, Rosario AS, Gerken M, et al (2002) Risk factors for lung cancer among nonsmoking women. Int J Cancer 100: 706–713. 12209611 10.1002/ijc.10549

[37] Turner MC, Chen Y, Krewski D, Calle EE, Thun MJ (2007) Chronic obstructive pulmonary disease is associated with lung cancer mortality in a prospective study of never smokers. Am J Respir Crit Care Med 176: 285–290. 17478615 10.1164/rccm.200612-1792OC

[38] Wu AH, Fontham ET, Reynolds P, Greenberg RS, Buffler P, et al (1995) Previous lung disease and risk of lung cancer among lifetime nonsmoking women in the United States. Am J Epidemiol 141: 1023–1032. 7771438 10.1093/oxfordjournals.aje.a117366

[39] Wu AH, Yu MC, Thomas DC, Pike MC, Henderson BE (1988) Personal and family history of lung disease as risk factors for adenocarcinoma of the lung. Cancer Res 48: 7279–7284. 3191498

[40] Schabath MB, Delclos GL, Martynowicz MM, Greisinger AJ, Lu C, et al (2005) Opposing effects of emphysema, hay fever, and select genetic variants on lung cancer risk. Am J Epidemiol 161: 412–422. 15718477 10.1093/aje/kwi063

[41] Brownson RC, Alavanja MC (2000) Previous lung disease and lung cancer risk among women (United States). Cancer Causes Control 11: 853–858. 11075875 10.1023/a:1008999202040

[42] Liang H, Guan P, Yin Z, Li X, He Q, et al (2009) Risk of lung cancer following nonmalignant respiratory conditions among nonsmoking women living in Shenyang, Northeast China. J Womens Health (Larchmt) 18: 1989–1995. 20044861 10.1089/jwh.2008.1355

[43] Mayne ST, Buenconsejo J, Janerich DT (1999) Previous lung disease and risk of lung cancer among men and women nonsmokers. Am J Epidemiol 149: 13–20. 9883789 10.1093/oxfordjournals.aje.a009722

[44] Osann KE, Lowery JT, Schell MJ (2000) Small cell lung cancer in women: risk associated with smoking, prior respiratory disease, and occupation. Lung Cancer 28: 1–10. 10704703 10.1016/s0169-5002(99)00106-3

[45] Jia-chun L, Lu-yuan S, Zhong-liang W (2003) Case-Control Study of Human Lung Cancers in Guangzhou. CHINA J CANCER PREV TREAT 10: 673–676.

[46] Koshiol J, Rotunno M, Consonni D, Pesatori AC, De Matteis S, et al (2009) Chronic obstructive pulmonary disease and altered risk of lung cancer in a population-based case-control study. PLoS One 4: e7380. 19812684 10.1371/journal.pone.0007380 PMC2753644

[47] Yong H, Hui X, An-Hui W, Yun-Jie W (2005) Chronic obstructive pulmonary diseases are risk factors of lung cancer. Journal of The Fourth Military Medical University 26: 146–149.

[48] Ko YC, Lee CH, Chen MJ, Huang CC, Chang WY, et al (1997) Risk factors for primary lung cancer among non-smoking women in Taiwan. Int J Epidemiol 26: 24–31. 9126500 10.1093/ije/26.1.24

[49] Wang XR, Yu IT, Chiu YL, Qiu H, Fu Z, et al (2009) Previous pulmonary disease and family cancer history increase the risk of lung cancer among Hong Kong women. Cancer Causes Control 20: 757–763. 19169896 10.1007/s10552-008-9289-4

[50] Neuberger JS, Mahnken JD, Mayo MS, Field RW (2006) Risk factors for lung cancer in Iowa women: implications for prevention. Cancer Detect Prev 30: 158–167. 16581199 10.1016/j.cdp.2006.03.001 PMC1876736

[51] Wu-Williams AH, Dai XD, Blot W, Xu ZY, Sun XW, et al (1990) Lung cancer among women in north-east China. Br J Cancer 62: 982–987. 2257230 10.1038/bjc.1990.421 PMC1971561

[52] Brenner AV, Wang Z, Kleinerman RA, Wang L, Zhang S, et al (2001) Previous pulmonary diseases and risk of lung cancer in Gansu Province, China. Int J Epidemiol 30: 118–124. 11171871 10.1093/ije/30.1.118

[53] Miao Z, Ya-Chen A (2009) The risk factors of lung cancer in the rural area of Tangshan, China. Chinese Journal of Coal Industry Medicine 12: 125–127.

[54] Wang SY, Hu YL, Wu YL, Li X, Chi GB, et al (1996) A comparative study of the risk factors for lung cancer in Guangdong, China. Lung Cancer 14 Suppl 1: S99–S105. 8785673 10.1016/s0169-5002(96)90215-9

[55] Brenner DR, McLaughlin JR, Hung RJ (2011) Previous Lung Diseases and Lung Cancer Risk: A Systematic Review and Meta-Analysis. PLoS One 6: e17479. 21483846 10.1371/journal.pone.0017479 PMC3069026

[56] Ardies CM (2003) Inflammation as cause for scar cancers of the lung. Integr Cancer Ther 2: 238–246. 15035887 10.1177/1534735403256332

[57] Brody JS, Spira A (2006) State of the art. Chronic obstructive pulmonary disease, inflammation, and lung cancer. Proc Am Thorac Soc 3: 535–537. 16921139 10.1513/pats.200603-089MS

[58] Young RP, Hopkins RJ, Whittington CF, Hay BA, Epton MJ, et al (2011) Individual and cumulative effects of GWAS susceptibility loci in lung cancer: associations after sub-phenotyping for COPD. PLoS One 6: e16476. 21304900 10.1371/journal.pone.0016476 PMC3033394

[59] Schwartz AG, Prysak GM, Bock CH, Cote ML (2007) The molecular epidemiology of lung cancer. Carcinogenesis 28: 507–518. 17183062 10.1093/carcin/bgl253

[60] Schwartz AG, Ruckdeschel JC (2006) Familial lung cancer: genetic susceptibility and relationship to chronic obstructive pulmonary disease. Am J Respir Crit Care Med 173: 16–22. 16141445 10.1164/rccm.200502-235PP PMC2662980

[61] Engels EA, Wu X, Gu J, Dong Q, Liu J, et al (2007) Systematic evaluation of genetic variants in the inflammation pathway and risk of lung cancer. Cancer Res 67: 6520–6527. 17596594 10.1158/0008-5472.CAN-07-0370

[62] Molfino NA (2007) Current thinking on genetics of chronic obstructive pulmonary disease. Curr Opin Pulm Med 13: 107–113. 17255800 10.1097/MCP.0b013e328013e97d

[63] Littman AJ, Thornquist MD, White E, Jackson LA, Goodman GE, et al (2004) Prior lung disease and risk of lung cancer in a large prospective study. Cancer Causes Control 15: 819–827. 15456995 10.1023/B:CACO.0000043432.71626.45

[64] Purdue MP, Gold L, Jarvholm B, Alavanja MC, Ward MH, et al (2007) Impaired lung function and lung cancer incidence in a cohort of Swedish construction workers. Thorax 62: 51–56. 16928722 10.1136/thx.2006.064196 PMC2111275

[65] Wasswa-Kintu S, Gan WQ, Man SF, Pare PD, Sin DD (2005) Relationship between reduced forced expiratory volume in one second and the risk of lung cancer: a systematic review and meta-analysis. Thorax 60: 570–575. 15994265 10.1136/thx.2004.037135 PMC1747470

[66] Pierrou S, Broberg P, O'Donnell RA, Pawlowski K, Virtala R, et al (2007) Expression of genes involved in oxidative stress responses in airway epithelial cells of smokers with chronic obstructive pulmonary disease. Am J Respir Crit Care Med 175: 577–586. 17158281 10.1164/rccm.200607-931OC

[67] Borczuk AC, Powell CA (2007) Expression profiling and lung cancer development. Proc Am Thorac Soc 4: 127–132. 17202302 10.1513/pats.200607-143JG

[68] Santillan AA, Camargo CJ, Colditz GA (2003) A meta-analysis of asthma and risk of lung cancer (United States). Cancer Causes Control 14: 327–334. 12846363 10.1023/a:1023982402137

[69] Schabath MB, Gorlova OY, Spitz MR (2006) Re: “Cancer mortality among US men and women with asthma and hay fever”. Am J Epidemiol 163: 394–395, 395–396. 16371513 10.1093/aje/kwj065

[70] Littman AJ, Jackson LA, Vaughan TL (2005) Chlamydia pneumoniae and lung cancer: epidemiologic evidence. Cancer Epidemiol Biomarkers Prev 14: 773–778. 15824142 10.1158/1055-9965.EPI-04-0599

[71] Zeki AA, Schivo M, Chan AL, Hardin KA, Kenyon NJ, et al (2010) Geoepidemiology of COPD and idiopathic pulmonary fibrosis. J Autoimmun 34: J327–J338. 20018478 10.1016/j.jaut.2009.11.004

