# Supplementary material for: Association between Chronic Obstructive Pulmonary Disease and Lung Cancer: A Case-Control Study in Southern Chinese and a Meta-Analysis
Source: PLoS One. 2012 Sep 28;7(9):e46144. doi: 10.1371/journal.pone.0046144 (PMC3460937; doi:10.1371/journal.pone.0046144)
Supplement: Protocol S1 — Additional details of methods. (DOC) [file pone.0046144.s001.doc]

**Online Data Supplement**

**Additional details of methods**

**1. Criteria or definitions of lung cancer and other lung diseases**

Lung cancer was diagnosed according to standard clinical criteria with pathologic confirmation from surgery, biopsy, or cytology samples. Main analyses included all primary lung cancer cases regardless of histological type.

The COPD diagnosis was according to the global initiative for chronic obstructive lung disease b**ased on medical history, chest radiographic findings, physical examination and spirometric data. Those individuals who had these chronic airway symptoms and signs such as chronic cough, dyspnea, sputum production, wheezing, and chronic airway obstruction that was defined as a FEV1/forced vital capacity (FVC) of <70% after inhalation of** 400μg **salbutamol were defined to have been pre-existing COPD. For other diseases like** emphysema, chronic bronchitis or asthma, they were diagnosed by **chest radiographic findings, physical examination, clinical symptoms and assisting exanimation like spirometry.**

2. **Definitions of co-variables**

Those participants who had smoked <100 cigarettes in their lifetime were defined as never smokers; otherwise, they were classified as ever smokers. Ever smokers who had quit for >1 year before enrollment were considered former smokers, and the remaining smokers were defined as current smokers. Pack year smoking was divided into three categories: 0 pack per year, <20 packs per year and ≥20 packs per year. Similarly, participants who had consumed alcoholic beverages at least once a week for ≥1 year previously were defined as ever drinkers, and the remaining drinkers as never drinkers. Ever drinkers who had quit drinking for ≥1 year previously were defined as former drinkers, and the others were defined as current drinkers. This study used the BMI cutoff points suggested by Cooperative Meta-Analysis Group of Working Group on Obesity in China [5]. Subjects whose BMI was ≤23.9 kg/m^2^ were categorized as being underweight or normal body weight, subjects whose BMI was from 24.0 to 27.9 kg/m^2^ were defined as being overweight, those who have a BMI ≥28.0 kg/m^2^ were categorized as being obese. Those who have any first or second-degree relative (or both) with a history of cancer were defined as ‘‘Yes,’’ and the remaining subjects as ‘‘No.’’

**References:**

[1] Rabe KF, Hurd S, Anzueto A, Barnes PJ, Buist SA, Calverley P, Fukuchi Y, Jenkins C, Rodriguez-Roisin R, van Weel C, Zielinski J. Global strategy for the diagnosis, management, and prevention of chronic obstructive pulmonary disease: GOLD executive summary. Am J Respir Crit Care Med 2007; 176: 532-555.

[2]. Standardization of spirometry, 1994 update. American thoracic society. Am J Respir Crit Care Med 1995;152:1107-1136.

[3]. Pellegrino R, Viegi G, Brusasco V, Crapo RO, Burgos F, Casaburi R, Coates A, van der Grinten CP, Gustafsson P, Hankinson J, Jensen R, Johnson DC, MacIntyre N, McKay R, Miller MR, Navajas D, Pedersen OF, Wanger J. Interpretative strategies for lung function tests. Eur Respir J 2005;26:948-968.

[4]. Zheng J, Zhong N. Normative values of pulmonary function testing in chinese adults. Chin Med J (Engl) 2002;115:50-54.

[5] Zhou BF. Predictive values of body mass index and waist circumference for risk factors of certain related diseases in Chinese adults--study on optimal cut-off points of body mass index and waist circumference in Chinese adults. Biomed Environ Sci 2002; 15: 83-96.
