# Supplementary material for: Association between Chronic Obstructive Pulmonary Disease and Lung Cancer: A Case-Control Study in Southern Chinese and a Meta-Analysis
Source: PLoS One. 2012 Sep 28;7(9):e46144. doi: 10.1371/journal.pone.0046144 (PMC3460937; doi:10.1371/journal.pone.0046144)
Supplement: Table S1 — Characteristics of 35 studies on the association between COPD and lung cancer. (DOC) [file pone.0046144.s002.doc]

Table S1 Characteristics of 35 studies on the association between COPD and risk of lung cancer.

| **Study** | **Year of publication** | **Ethnicity** | **Cases**  **number** | **Control**  **Number** | **Control**  **Type** | **Study type** | **Diagnosis**  **of COPD** | **Diagnosis of lung**  **cancer** |
| --- | --- | --- | --- | --- | --- | --- | --- | --- |
| Schwartz et al. | 2009 | Caucasian | 562 | 564 | Community | CC | Physian | Pathology |
| Annie et al. | 2002 | Caucasian | 118 | 235 | Hospital | CC | Questionare | Pathology |
| Young et al. | 2009 | Caucasian | 446 | 484 | Community | CC | Spirometry | Pathology |
| Yang et al. | 2008 | Caucasian | 1856 | 1585 | Community | CC | Physian | Pathology |
| Margaret et al. | 2008 | Caucasian | 725 | 615 | Hospital | CC | Physian | Pathology |
| Juan et al. | 2007 | Caucasian | 23 | 1143 | Community | CH | Spirometry | Pathology |
| David et al. | 2008 | Caucasian | 99 | 3539 | Community | CH | Spirometry | CT |
| Antonio et al. | 2006 | Caucasian | 5263 | 20000 | Community | CC | Physian | Physian |
| Wang et al. | 1996 | Asian | 390 | 390 | Community | CC | Spirometry | Pathology |
| Jill et al. | 2009 | Caucasian | 1934 | 2108 | Community | CC | Physian | Pathology |
| Satoko et al. | 2009 | Asian | 245 | 813 | Community | CC | Spirometry | CT |
| Papi et al. | 2004 | Caucasian | 86 | 54 | Hospital | CC | Spirometry | Pathology |
| Alison et al. | 2009 | Caucasian | 459 | 483 | Community | CC | Physian | Physian |
| Carol et al. | 2008 | African | 491 | 497 | Community | CC | Physian | Pathology |
| John et al. | 2006 | Caucasian | 413 | 614 | Community | CC | Physian | Pathology |
| Lv et al. | 2003 | Asian | 445 | 445 | Hospital | CC | Spirometry | Pathology |
| Carlotta et al. | 2008 | Asian | 218 | 436 | Hospital | CC | Spirometry | Pathology |
| Osann et al. | 1991 | Caucasian | 184 | 184 | Hospital | CC | Spirometry | Pathology |
| Ross et al. | 2000 | Caucasian | 676 | 700 | Community | CC | Spirometry | Pathology |
| Susan et al. | 1999 | Caucasian | 437 | 437 | Community | CC | Questionare | Pathology |
| Alina et al. | 2001 | Asian | 886 | 1765 | Community | CC | Questionare | Chest X ray |
| Wang et al. | 2009 | Asian | 212 | 292 | Community | CC | Questionare | Pathology |
| Ko et al. | 1997 | Asian | 105 | 105 | Hospital | CC | Questionare | Pathology |
| Liang et al. | 2009 | Asian | 226 | 279 | Hospital | CC | Questionare | Pathology |
| Osann et al. | 2000 | Caucasian | 98 | 204 | Community | CC | Questionare | Pathology |
| Wu et al. | 1995 | Caucasian | 412 | 1253 | Community | CC | Questionare | Pathology |
| Maldonado et al. | 2010 | Caucasian | 64 | 377 | Community | CC | Spirometry | CT |
| Han et al. | 2005 | Asian | 248 | 263 | Hospital | CC | Questionare | Pathology |
| Yu et al. | 2000 | Asian | 1312 | 1312 | Community | CC | Questionare | Pathology |
| Wang et al. | 1995 | Asian | 390 | 390 | Hospital | CC | Questionare | Pathology |
| Shi et al. | 2007 | Asian | 173 | 173 | Hospital | CC | Questionare | Chest X ray |
| Zhou et al. | 2009 | Asian | 79 | 79 | Hospital | CC | Questionare | Pathology |
| Schabath et al. | 2004 | Caucasian | 1553 | 1375 | Hospital | CC | Questionare | Pathology |
| David et al. | 1986 | Caucasian | 113 | 113 | Hospital | CH | Spirometry | Pathology |
| Yang et al. | unpulished | Asian | 1069 | 1132 | Community | CC | Questionare | Pathology |

CC, case control study; CH, cohort study.
