# Supplementary figures and images for: Association between Chronic Obstructive Pulmonary Disease and Lung Cancer: A Case-Control Study in Southern Chinese and a Meta-Analysis
Source: PLoS One. 2012 Sep 28;7(9):e46144. doi: 10.1371/journal.pone.0046144 (PMC3460937; doi:10.1371/journal.pone.0046144)

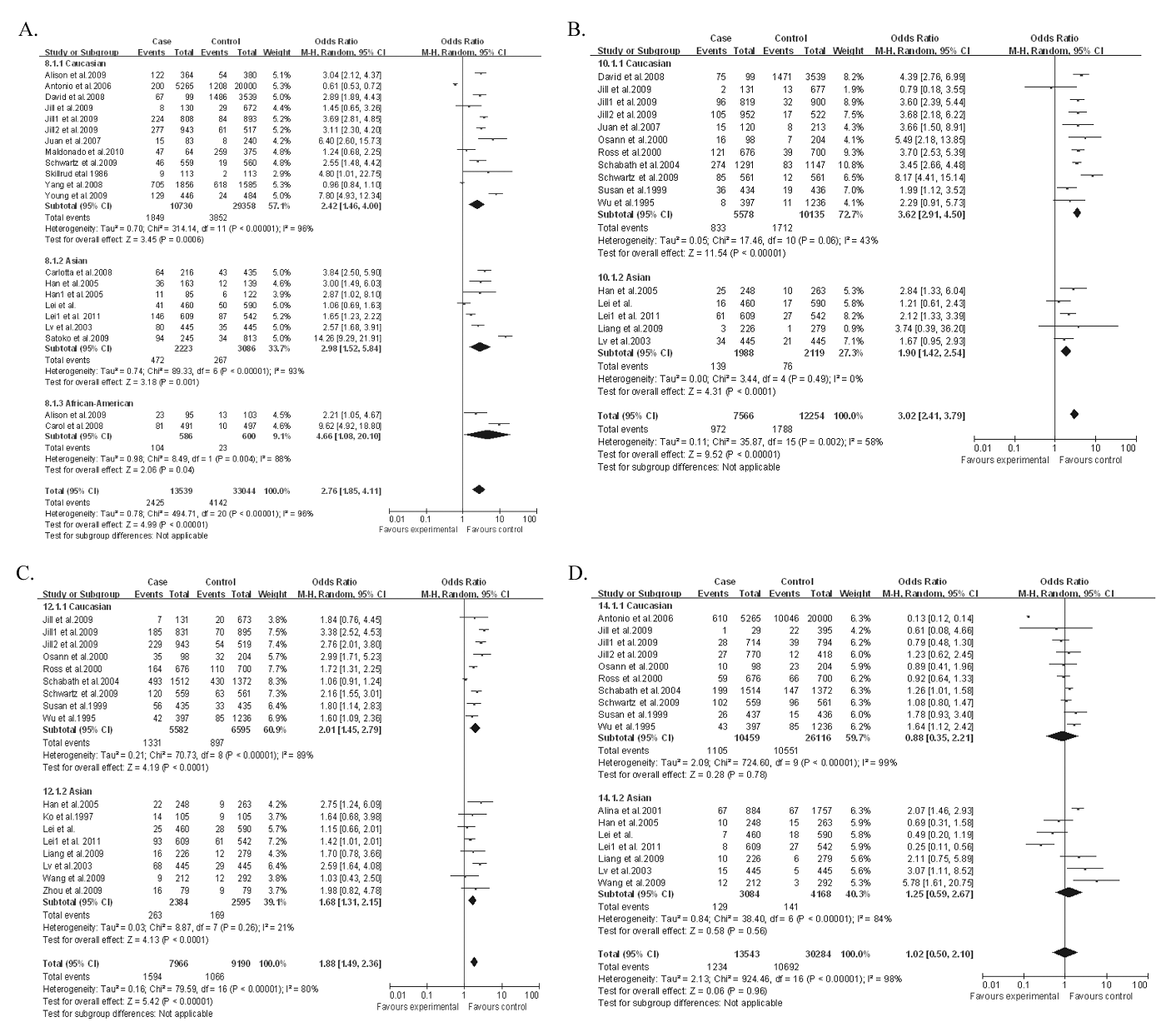

Supplement: Figure S1 — A.Pooled effect estimates of the lung cancer risk associated with a previous history of COPD, separated by ethnicity and overall. B. Pooled effect estimates of the lung cancer risk associated with a previous history of emphysema, separated by ethnicity and overall. C. Pooled effect estimates of the lung cancer risk associated with a previous history of chronic bronchitis, separated by ethnicity and overall. D. Pooled effect estimates of the lung cancer risk associated with a previous history of asthma, separated by ethnicity and overall. (TIF) [file pone.0046144.s003.tif]

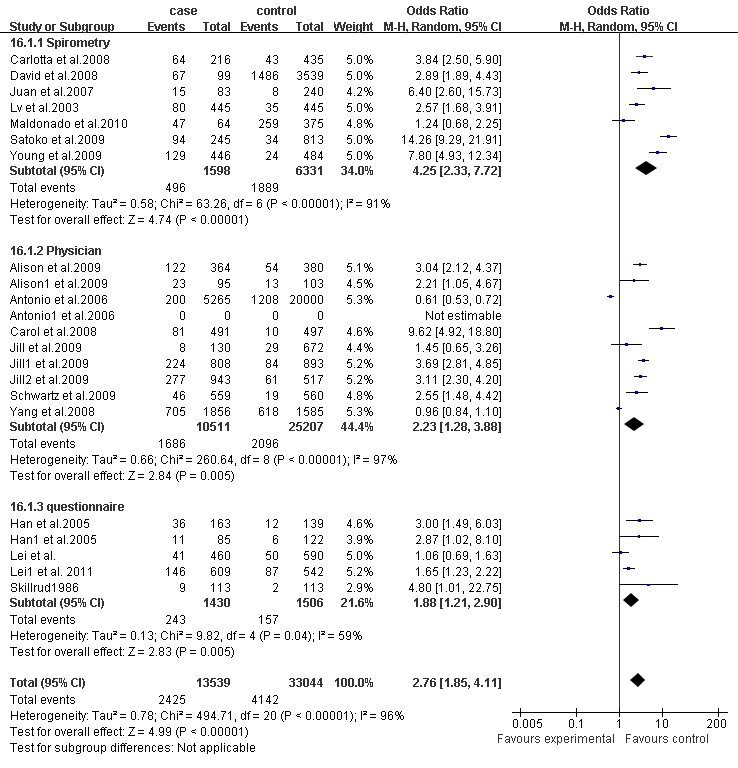

Supplement: Figure S2 — Pooled effect estimates of the lung cancer risk associated with a previous history of COPD, stratified by method of COPD diagnosis and overall (sensitivity analysis). (TIF) [file pone.0046144.s004.tif]
